# AI-informed healthcare pedagogy: a review of the literature and application to pedagogical practice informed by case studies from dental education

**DOI:** 10.1038/s41415-026-9954-6

**Published:** 2026-09-11

**Authors:** Nilufar Ahmed

**Affiliations:** Bristol Dental School, University of Bristol, Bristol, United Kingdom

## Abstract

Artificial intelligence (AI) applications in healthcare continue to grow at a rapid pace. Healthcare education is struggling to effectively integrate the manifold applications and opportunities presented by AI to education. Concerns and fears around negative impacts on student learning and potential for misuse alongside a paucity of guidance has led to a sluggish response across much of healthcare education. This paper offers a short review of the applications of AI in healthcare including clinical dentistry and makes suggestions for bridging the gap between education and practice so that educators can better prepare students for their professional careers. This paper argues that open, reflective classroom discussions around ethical concerns and climate impacts of AI are as important as the positive clinical applications to endow students with the knowledge to make decisions that align with social justice and social accountability. Two widely used assessments in dental and wider healthcare education − reflective assessments and evidence-based practice projects − are explored as case studies to develop an updated, AI-informed approach to teaching. Drawing on pedagogical literature, the author offers suggestions for educators to support thoughtful and ethical integration of AI within a student's learning repertoire so that AI becomes supplementary to, and not a substitute for, learning.

## Key points

- Artificial intelligence (AI) is being increasingly adopted in clinical practice and applied to support patient communication, diagnostics and treatment.
- Healthcare education has been slower to integrate AI into teaching and learning for students.
- Reflective assessments and evidence-based practice projects build critical thinking and self-awareness. Suggestions to integrate AI into these are made.

## Introduction

The rapid evolution of artificial intelligence (AI) technologies offers significant benefits for healthcare providers and patients.^1^^,^^2^^,^^3^^,^^4^ Availability of large-scale datasets such as electronic health records and digital imaging banks have expedited AI use across healthcare settings, from aiding diagnosis across a range of health conditions^5^^,^^6^^,^^7^ to supporting treatment planning. Clinical dentistry has seen AI applications result in increased effectiveness of interventions.^8^^,^^9^ As well as streamlining clinical care,^10^^,^^11^ AI has the potential to enhance patient experience and improve communication through personalised oral health advice.^12^^,^^13^ A plethora of reviews on applications of AI in healthcare^14^^,^^15^^,^^16^ describe the functionality and range of AI applications. That is not the scope of this review. This paper does not seek to replicate these reviews or delve into clinical applications, rather to review these findings in relation to healthcare pedagogy to prepare students for AI in clinical practice and build effective pedagogical practice. In doing so, this paper offers insight into how AI can be introduced into healthcare education to ensure students benefit from the opportunities AI promises without compromising on depth of learning; and integrates considerations for social justice and ethical responsibilities expected across healthcare disciplines.

Clinical advantages of AI have ensured its adoption into clinical settings at a swifter rate than into corresponding educational settings. Applications of AI extend to dental and medical education and research,^17^^,^^18^ and healthcare curricula must rapidly respond to the growing use of AI in clinical practice. It is imperative educators consider ways to integrate AI into teaching so students can be guided in not just effective use, but also the ethics of AI.^19^^,^^20^ There have been warnings of AI exacerbating health inequalities,^21^^,^^22^ and thus integrating AI ethics across the dental curriculum must be considered across the teaching body. Katznelson and Gerke^23^ have identified key domains to integrate AI ethics in medical education. These domains of informed consent, bias, safety, transparency, patient privacy, and allocation of services, apply across the undergraduate dental curriculum. Concerns have been raised about a lack of transparency, limiting human effort,^24^ reducing students' ability to think critically,^25^ impacting ethics and professionalism,^26^ reinforcing biases and discrimination,^27^ and wider sustainability and climate impacts of AI which contribute to expansion of global health disparities.^28^ Climate impacts of dentistry^29^ are increasingly explored in dental education; however, this cannot be seen as separate to the enormous climate impacts of AI usage across healthcare. While this expansive discussion is beyond the scope of this paper, it highlights the urgency of integrating AI into dental education.

Studies exploring resistance to introducing AI chatbots in dental education have identified a range of barriers, from faculty resistance to change^30^ and lack of training among educators,^25^ to gaps in knowledge risking inaccuracies in information returned,^31^ fears of misuse, and concerns around plagiarism.^32^

AI chatbot functionality in personalising information has been recognised for its potential for patient engagement; and this personalisation extends to tailoring educational content for students to align with their personal learning preferences. Research has found AI can increase student engagement and learning.^33^ Many Gen Z students have had technology as a constant part of their lives,^34^ and AI is already part of their established learning landscape. AI can assist students' learning by providing additional prompts and answering questions to aid deeper learning.^35^

Studies show students exhibit lower levels of anxiety when engaging with chatbots compared to traditional learning materials.^36^ The reduction in anxiety may arise from feeling less nervous asking questions to a chatbot compared to asking questions in a class environment where students may worry about how they are perceived.^37^

It is not just students whose mental health could be positively impacted by embracing AI, it can also ease pressures on faculty staff – something important to consider as academic and professional services staff are reporting unprecedent levels of burnout^38^ across higher education. Deploying AI tools to streamline workloads in some areas can create time and space to focus on building deeper connections in other aspects of education^39^ where AI simply cannot substitute the relational learning experience. Community engaged education and social accountability are core themes of the modern dental and wider healthcare curricula, yet these areas require time to build relationships and understanding of diverse patient needs for both educators and students.^40^ It is critical to invest time in this given evidence that overstretched staff and lack of capacity for delivering this type of teaching appropriately can adversely affect student wellbeing and learning.^41^

This narrative review^42^ has summarised the burgeoning application of AI in professional healthcare settings. This field is rapidly growing, and the literature will have evolved by time of publication. The paper will now focus on ways to bridge the gap in application between healthcare practice and healthcare education. There is limited consensus or guidance on integrating AI into medical^43^ and dental education.^44^ However, learning outcomes for both the General Dental Council's *Safe practitioner framework* and the General Medical Council's *Outcomes for graduates* offer ample opportunities to facilitate discussions around AI use for learning, transparency, and critical engagement.

Below, two common assessments in healthcare education, including dentistry, will be explored as case studies with a view towards creating an AI-informed approach that has application for all professional healthcare educators. Suggestions are made for where and how to integrate AI to foster critical reflection and critical evaluation. The first is reflective assessments where students could convincingly use AI tools to generate rapid reflective responses on their behalf. Using AI to generate reflections would negate the methodical and deliberately slow nature of personal reflection which is fundamental to the cognitive processing that can lead to change. Guidance is provided on ways to support and deepen student reflection even if AI is used. The second case study explores the strengths and limitations of AI within evidence-based projects designed to encourage students to critically evaluate research.

## Reflective assessments

Critical thinking is a fundamental attribute of the healthcare provider.^45^ Reflective writing increases critical thinking, clinical reasoning, and problem solving among students,^46^ as well as developing confidence and authenticity in professional identity. It builds awareness of personal ways of working, personal assumptions and biases, and increases understanding of wider sociocultural factors impacting patient lives.^47^ This section discusses the importance of reflective assessments across healthcare education and examines literature-informed approaches to applying AI in reflective assessments. Options for ways educators can support the development of not only reflective skills but also support the building of confidence and connection with students are proposed.

### What are reflective assessments?

The ability to think reflectively is an essential skill in the health professions.^48^ The exercise of actively reflecting is widely applied within foundational health professional education.^49^ Various models and theories of reflection^50^ have been widely used across healthcare education. Reflective essays apply these theories and typically encourage students to share situational learning experiences and discuss how this might impact future behaviour. This process can result in ‘transformative knowledge' building where new knowledge emerges that can lead to changes in clinician behaviour.^51^

Reflective writing can increase empathy,^52^ thus mitigating against the decline in empathy often noted during foundational training.^53^ Reflective practice is also linked with heightened resilience and improved mental health for healthcare professionals.^54^ Given the high levels of poor mental health among dental professionals^55^ critical reflection is a vital skill for healthcare professionals wellbeing.

### AI applications in reflective assessments

Despite the many advantages of reflective practice across health professions, reflection does not always come easily to students; accounts can be superficial and descriptive in nature.^56^ This has been attributed to the clinical biomedical model of health being dissonant to reflective practice which encourages non-clinical thinking.^57^ Students may feel pressured to write what they feel is expected of them, rather than engaging in critical reflection*.*^58^

AI chatbots may offer a resource for students struggling with reflection to input a real-life encounter and retrieve reflective responses. With research finding dental instructors attributing AI-generated reflections to student's own work^49^ it might be impossible to differentiate responses generated by AI from student reflections. Rather than challenging the use of AI out of fears of it replacing student effort, our focus as educators must be on ensuring evidenceable delivery of learning outcomes, in this case developing critical reflection skills. Perhaps AI might generate an adequate response in the first instance, but educators can help to probe, challenge, develop, and deepen responses whatever their origin. One option for this might be to involve personal tutors.

### Developing connections as a counter to AI

All UK undergraduate students have access to personal tutors they meet with across the academic year. Creating a structured one-to-one tutoring session to discuss the reflective assessment can support personal tutors who may feel unsure in how to structure personal tutoring.^59^ The tutor could ask open questions building on the assessment to facilitate deeper reflections and understanding and thus directly demonstrate the value of reflection. Guidance and probing questions should be circulated to all personal tutors involved in these discussions to ensure they are confident in reflective processes and questions.

Having clarity in the expectation of the content of a personal tutoring session is beneficial to both tutor and student^60^ and planning to review the reflection can facilitate deeper conversations. Research with medical students has found personal tutoring can deepen reflection.^61^ It can help create a safe learning space for students who may find large clinical spaces stressful. Learning in a clinical environment for healthcare professions such as dentistry can be stressful, causing negative impacts on students' physical and mental health.^62^ Dental students may feel insecure regarding their contributions, which in turn, may present as a barrier to learning.^63^ A one-to-one review can mitigate against insecurities with opportunities for the tutor to encourage, clarify, and acknowledge positive practice as well as guide deeper learning. For tutors, a structured session to review the reflective assessment can offer scaffolding for the tutor in their role.^64^ They can use a range of provided probes and open questions to further explore student reflections. This process not only builds critical thinking skills for the student but also deepens rapport and relationship with the personal tutor, a factor known to impact the educational experience of students.^65^

This integration of AI into reflective assessments can mimic a recognised benefit of AI – the personalisation of responses – but coming from a familiar source within the educational setting, it can deepen the connection and community in learning spaces. The reflective discussions may also open other avenues of educational or pastoral discussion that can benefit the student in their learning environment. This relational aspect cannot be offered by AI.

## Evidence-based practice

The second AI-informed dental education case study explored here is evidence-based practice (EBP) projects. EBP is considered the gold standard of clinical practice^66^ and has proven demonstrable benefits on treatment quality and outcomes.^67^ Hence, EBP projects are an important feature of undergraduate dental programs^68^ supporting students to learn critical analysis skills they can take forward beyond their education.^69^

This section explores how AI can be applied to streamline EBP projects to liberate time to focus in greater depth on others. Additionally, it is proposed that working with students to co-create guidance on application of AI in EBP projects aligns with wider educational aspirations of more inclusive and decolonial pedagogical practice^70^ in healthcare education.

### What are EBP projects?

Traditional EBP projects typically apply a fivestep model of:

- Ask
- Acquire
- Appraise
- Apply
- Assess.^71^

In such projects, students may formulate or be allocated a research question, conduct literature search, critically appraise research, synthesise findings, and make recommendations. EBPs are often undertaken in pairs or small groups to develop collaboration and teamworking skills.

### AI applications in evidence-based projects

Integrating AI into EBP projects may facilitate learning for students and have positive outcomes through personalising learning,^72^ and help students more efficiently locate relevant research articles. Depending on prompts used, AI programs can produce effective summaries of articles and highlight methodological details (e.g., study design, outcomes, limitations), to help students become familiar with structure and content and scaffold learning^73^ before undertaking deep appraisal. Spending less time retrieving information allows more time for appraisal and synthesis of data.

However, to be most effective educationally, AI must be integrated in a way which supports rather than substitutes students' engagement and critical thinking. One approach might be to encourage students to reflect on the advantages and disadvantages of both methods. To follow a traditional model for the project in the first instance, explicitly avoiding AI. Students will note difficulties, questions that arose, decision processes for inclusion, discussions among the team, and time taken. Once this process has been completed, input the question into AI software and see what is returned, from initial prompts, to refining questions and the questions returned from the AI to hone its process. Students could compare AI-generated summaries with their own analysis of article abstracts; and justify differences between both and reflect on biases that might be evident in AI suggestions. While AI can be efficient in reducing time in undertaking an evidence summary, time must be taken to learn the tools to become effective in asking and refining questions^74^ and this could be built into a revised EBP project.

Students could explore different ways of building AI into their EBP, for example use of prompts to explore methodological rigour, or to engage with AI as a conversational partner to facilitate their deeper analysis and reasoning, as opposed to simply generating a prepared response. These reflective discussions could offer a deeper understanding of the ethics of AI tools and an exploration of biases built into AI algorithms,^75^ which can help mitigate against automatically accepting the results returned by AI without critical interrogation.

This explicit disclosure and open interrogation of the benefits of both traditional and AI approaches can help move away from AI being viewed with suspicion or confusion, and lead to deeper dialogue around ethics and impact on learning. This can be further extended through open discussions with students after completion of a project around what guidance and/or restrictions on AI usage could be applied for future iterations of EBPs, especially when framed with the explicit goal of increasing learning.

### Co-creation with students as a way of developing inclusive teaching

Adopting AI thoughtfully and creatively into EBP projects through co-creation with students can support wider pedagogical aims for more inclusive learning. In co-creating curricula, students are considered partners in producing educational materials. Despite being relatively underexplored in dental education,^76^ co-creation is recognised as facilitating learning and reflective skills for both students and staff.^77^^,^^78^ Co-creating learning is evidenced to empower students and increase engagement in learning.^79^ A sense of ownership, autonomy and responsibility for education can result in deeply reflective and transformative learning experiences.^80^

Importantly, co-creation helps disrupt power hierarchies in education.^81^ This is especially relevant in dental education where attitudes to change and power remain fixed, as exhibited by traditional teaching methods and leadership demographics^82^ which can be barriers to decolonial efforts.^83^ Introducing co-creation via exploration of AI offers a novel way of disrupting these entrenched patterns while reintroducing human contact and connection as a corollary to balance the impersonal engagement intrinsic in AI.

## Conclusion

The widespread, accelerating application of AI in healthcare highlights the importance of curricula to become AI-informed, incorporating AI literacy, and addressing ethical issues in its usage.^84^ It is essential health educators embrace AI to be able to instruct students on how to use AI safely, critically, and ethically to support their learning, and to minimise risk to patient care.^85^ Integrating AI into dental education holds significant potential for enhancing learning. With pedagogical rigour and ethical safeguards, AI can act as a cognitive scaffold, offering structured prompts, rapid evidence retrieval, and adaptive feedback, but these features must be mediated through human judgement, critical reflection, and tutor engagement.

In addition to reviewing and distilling available literature on AI-informed pedagogy within healthcare education, this paper has also considered the application of an AI-informed approach to integrating AI tools into two different widely applied learning scenarios in healthcare education, reflective assessments and EBP. Consideration of existing literature informs us that for reflective assessments AI could be instructed to prompt students to delve deeper into their reasoning and ethical considerations, while tutors' probe beneath AI supported reflections to explore students' authentic learning and implications of reflections for behavioural change. In EBP, AI can accelerate information retrieval but must be positioned as a tool to support critical appraisal rather than replace it. In both cases, transparency, academic integrity, and tutor mediated interpretation are core to educational outcomes.

These case studies are offered as suggestions and educators are encouraged to consider ways of integrating AI into other academic assessments such as portfolio-based assessments. Assessment criteria on AI use should mirror marking criteria for non-AI assessments – rewarding students who critically interrogate AI outputs, identify limitations, and demonstrate deep engagement with primary sources.

AI is already transforming the educational landscape; however, the benefits cannot be harnessed for deeper learning without careful educational design, oversight, and critical engagement on the part of both learners and educators. Educators have a responsibility to upskill and invest in their own awareness of, and proficiency with, AI tools. Co-creation offers one approach that leverages shared learning from digital natives. Co-creation of guidance on permissible AI use (e.g., literature search versus writing of analysis), can help ensure AI supports rather than undermines learning. Guidance may require reflexive statements of AI use, where students disclose how AI influenced their process and justify their human judgement.

Ideally all educational institutions would have mandatory training for students and staff on general applications and implications (including legal and ethical) of AI use that would apply to all courses irrespective of discipline. However, educators waiting for institutional and/or professional body guidance on AI risk falling behind. Sufficient evidence and literature are available to begin to creatively and thoughtfully explore a more AI-informed approach to education. Existing guidance from UNESCO (United Nations Educational, Scientific and Cultural Organization)^86^^,^^87^ offers clear and balanced roadmaps for integrating AI into education and provide a fruitful baseline for all healthcare educators.

## Data Availability

All data supporting the findings of this study are available within the paper.
